# Real-Time On-Site Diagnosis of Quarantine Pathogens in Plant Tissues by Nanopore-Based Sequencing

**DOI:** 10.3390/pathogens11020199

**Published:** 2022-02-02

**Authors:** Luca Marcolungo, Alessandro Passera, Simone Maestri, Elena Segala, Massimiliano Alfano, Francesca Gaffuri, Giovanni Marturano, Paola Casati, Piero Attilio Bianco, Massimo Delledonne

**Affiliations:** 1 Department of Biotechnology, University of Verona, Strada Le Grazie 15, 37134 Verona, Italy; luca.marcolungo@univr.it (L.M.); simone.maestri@univr.it (S.M.); elena.segala@univr.it (E.S.); massimiliano.alfano@univr.it (M.A.); giovanni.marturano@univr.it (G.M.); 2 Department of Agricultural and Environmental Sciences—Production, Landscape, Agroenergy, University of Milano, Via Celoria 2, 20133 Milan, Italy; alessandro.Passera@unimi.it (A.P.); paola.casati@unimi.it (P.C.); piero.bianco@unimi.it (P.A.B.); 3 Servizio Fitosanitario Regione Lombardia Laboratorio Fitopatologico c/o Fondazione Minoprio, 22100 Minoprio, Italy; francesca_gaffuri_cnt@regione.lombardia.it; 4 Institute for Sustainable Plant Protection, National Research Council (IPSP-CNR), Strada delle Cacce, 73, 10135 Turin, Italy; 5 Genartis S.r.l., Via P. Mascagni 98, 37060 Castel D’Azzano, Italy

**Keywords:** plant pathogen, diagnostics, subspecies, nanopore sequencing, MinION

## Abstract

Rapid and sensitive assays for the identification of plant pathogens are necessary for the effective management of crop diseases. The main limitation of current diagnostic testing is the inability to combine broad and sensitive pathogen detection with the identification of key strains, pathovars, and subspecies. Such discrimination is necessary for quarantine pathogens, whose management is strictly dependent on genotype identification. To address these needs, we have established and evaluated a novel all-in-one diagnostic assay based on nanopore sequencing for the detection and simultaneous characterization of quarantine pathogens, using *Xylella fastidiosa* as a case study. The assay proved to be at least as sensitive as standard diagnostic tests and the quantitative results agreed closely with qPCR-based analysis. The same sequencing results also allowed discrimination between subspecies when present either individually or in combination. Pathogen detection and typing were achieved within 13 min of sequencing owing to the use of an internal control that allowed to stop sequencing when sufficient data had accumulated. These advantages, combined with the use of portable equipment, will facilitate the development of next-generation diagnostic assays for the efficient monitoring of other plant pathogens.

## 1. Introduction

Plant pathogens and the diseases they cause are severe threats to global food security, resulting in yield losses of up to 30% in major staple crops and requiring the use of large quantities of pesticides for pathogen and/or vector control [1,2,3]. The risk of new pathogens introduced by invasive species is a particular concern because endogenous plants have no natural defenses and are, therefore, highly susceptible [4]. The majority of such parasites are classified as quarantine pests according to the FAO-ICPP (International Plant Protection Convention) standards, and the risk of their spread has greatly increased throughout the world, mostly due to the rise in global trade and transport [5]. Furthermore, the effects of climate change allow such pathogens and their vectors to thrive in previously non-permissive environments [6,7]. The risk to food security and safety is exacerbated by the prevalence of monocultures, which in some cases are limited to a single genotype, providing a homogeneous genetic environment that can easily select for host-specialized crop pathogens.

The diseases caused by quarantine pathogens can be controlled, and even prevented, by fast, efficient, and inexpensive diagnostic methods that allow early detection and the deployment of countermeasures. Gold standard methods include the enzyme-linked immunosorbent assay (ELISA) to detect pathogen proteins, and the polymerase chain reaction (PCR), quantitative real-time PCR (qPCR), or loop-mediated isothermal amplification (LAMP) to detect pathogen nucleic acids. These are quicker and more reliable than traditional methods requiring pathogen cultures, but sample preparation and analysis can take a long time, and the results are not quantitative unless standards of known concentration are available. Most importantly, there is a trade-off between detection range and specificity. Assays that detect broadly conserved sequences or proteins have the greatest range, but a secondary pathogen-specific assay is then required to identify particular strains, subspecies, or pathovars. For example, the identification of subspecies usually involves multilocus sequence typing (MLST), in which end-point PCR products are sequenced and compared with reference strains in a multi-step protocol. Conversely, assays with the desired specificity fail to capture a broad picture of the pathogens in a given sample and, therefore, suffer from ascertainment bias [8]. The effective management of quarantine pathogens, particularly those subject to international legislation [9], requires diagnostic procedures that are, at the same time, both broad and specific, as well as fast, sensitive, reliable, quantitative, and inexpensive.

The need for obtaining further insights, other than the detection of pathogen absence/presence, is exemplified by *Pseudomonas syringae*, a bacterial pathogen species that causes necrotic spots on the leaves or cankers on the trunks of many plants [10]. The species is divided into multiple pathovars based on genomic features and host range, but only two of them are regulated by quarantine measures in Europe [11]. Another example is Bois Noir disease in grapevine, where different strains of the quarantine pathogen *Candidatus* Phytoplasma solani are associated with different epidemiological cycles and different secondary hosts [12]. Finally, the accurate identification of subspecies is necessary for the management of *Xylella fastidiosa* (XF), which is a commensal on most of its > 500 plant hosts [13], but when infecting some hosts, it becomes a devastating pathogen [14]. *Xylella fastidiosa* can be subcategorized in five subspecies, two of which have only been proposed (subsp. *morus* and *sandyi*), while the other three are widely recognized and supported [15,16]. Of these three subspecies, *X. fastidiosa* subsp. *fastidiosa* (XFF) causes Pierce’s disease in grapevine [17], *X. fastidiosa* subsp. *multiplex* (XFM) causes different diseases of stone fruits, citrus, and coffee plants [14], and *X. fastidiosa* spp. *pauca* (XFP) is currently notorious for the epidemic on the olive trees in the Apulia region of Italy. 

All-in-one diagnostic tests that detect infections and simultaneously determine the pathogen genotypes in a quantitative manner are highly desirable because they allow earlier interventions to prevent or limit the spread of infection, and can, thus, shorten the quarantine periods for goods in transit. Approaches based on next-generation sequencing (NGS) are very promising because they make it possible to achieve the sensitive detection of pathogens while delivering the genomic sequence data necessary for quantitative genotype-specific identification, including emerging genetic variants [18]. For example, NGS has recently been used to detect phytopathogenic fungi (*Magnaporthe oryzae* and *Fusarium* spp.), oomycetes (*Phytophthora* spp.), bacteria (“*Candidatus* Liberibacter asiaticus”), and viruses (*Carrot yellow leaf virus*) [19]. Illumina sequencing was recently used to detect and identify particular subspecies of XF; although this method proved efficient, it involves the use of dedicated laboratory facilities and trained personnel [20]. The Oxford Nanopore Technologies (ONT) MinION device offers the same advantages as other NGS platforms but is also portable and inexpensive, allowing in-field deployment on a larger scale. ONT-based sequencing was shown suitable for the surveillance and identification of viral and bacterial pathogens that infect humans or plants [21,22,23,24], and can distinguish between single-nucleotide variants to resolve different isolates, strains, and subspecies [25,26,27]. Moreover, it has been widely used for in-field sequencing, including the monitoring of Zika virus infections, thus, highlighting its suitability for point-of-care testing [28,29,30,31,32,33,34]. Here, we describe the development and the evaluation of an ONT-based diagnostic assay for the detection and simultaneous characterization of quarantine pathogens, using *Xylella fastidiosa* as a case study. 

## 2. Results

**Figure 1:**
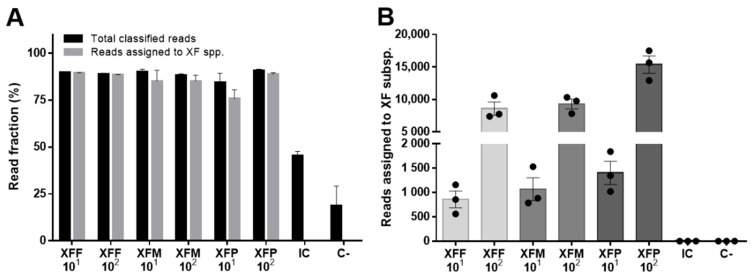
ONT sequencing identifies *X. fastidiosa* (XF) subspecies in samples spiked with DNA from individual subspecies. (**A**) For each condition, the bar graph shows the total number of reads with a BLAST hit (total classified reads) and the reads correctly assigned to each XF subspecies. (**B**) For each condition, the chart shows the number of reads assigned to each XF subspecies. Data are means ± SE (n = 3 technical replicates). XFF = XF subsp. *fastidiosa*; XFM = XF subsp. *multiplex*; XFP = XF subsp. *pauca*; IC = internal control; C^–^ = negative control.

We initially tested the ONT-based diagnostic assay for XF using samples comprising 10 ng/µL genomic DNA from the healthy potential host plant *Nerium oleander* (NO) alone as a negative control (C^–^) or spiked with 10^1^ or 10^2^ genome copies/µL of XFF, XFM, or XFP (Table 1). These concentrations correspond to the detection limits of traditional qPCR testing and a typical positive detection in infected plant tissue, respectively [8,35,36]. A ˜900-bp region of the *Xylella* gene encoding protein HL was amplified in triplicate from each sample with primers annealing to sequences conserved in each subspecies, and the resulting amplicons were used to generate a multiplex ONT sequencing library. ONT sequencing generated 312,629 reads in total, among which 133,399 (43%) could be demultiplexed and 128,318 (41%) were also PASS. This corresponded to means of 12,673 and 1346 reads from samples with 10^2^ and 10^1^ XF genome copies, respectively. The demultiplexed PASS reads were used as BLAST queries, and an average of 88.6% matched sequences in the NCBI nt database (Figure 1A). At both concentrations, the workflow identified the presence of XF reads only in the spiked samples and classified 96.3% of the reads as the correct subspecies (Figure 1A,B). The small number of reads from the C^–^ sample containing oleander DNA alone did not match any XF sequences (Figure 1A,B), confirming the ability of the assay to distinguish XF positive signals from a background of unrelated host DNA.

**Figure 2:**
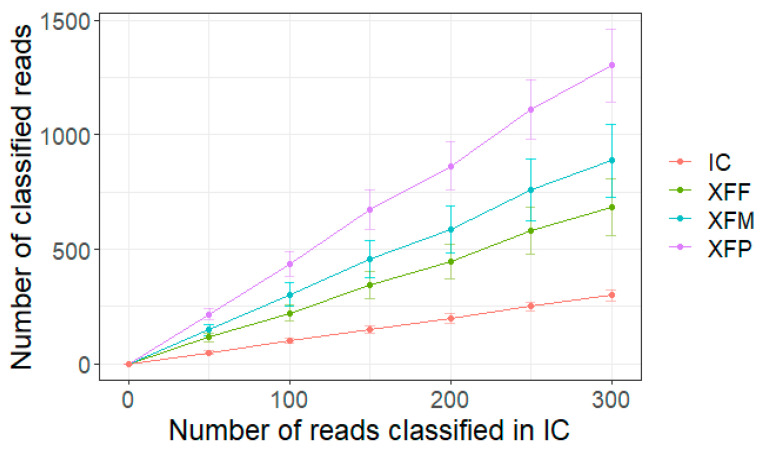
Production of *X. fastidiosa* (XF) sequencing reads in comparison to the internal control (IC) sample. The line graph shows the number of reads assigned to XF subspecies for samples with 10^1^ copies/µL spiked-in DNA when the number of reads assigned to the IC sample reached 50, 100, 150, 200, 250, and 300. Data are means ± SE (n = 3).

Along with the test samples, we also sequenced a known amount (3 ng) of an internal control sample (IC) consisting of an amplicon generated from a species unrelated to XF. The resulting amplicon concentration was at least 10-fold lower than the amplicons generated from the XF-spiked samples. This highly diluted IC sample was used to monitor when the amount of sequencing data was sufficient and the run could be stopped. When this point is reached, the presence of any XF in the test samples should have been detected. To assess the feasibility of this approach, the number of reads for each sample-barcode was monitored in a retrospective manner during the sequencing run. Figure 2 shows how many reads were attributed to each XF subspecies in the sample with the lowest concentration (10^1^ copies/µL) when approximately 50, 100, 150, 200, 250, and 300 reads on average were assigned to the IC sample barcode. A consistently higher number of reads was always assigned to 10^1^ copies of XF compared to the IC sample. For example, when 50 reads were assigned to the IC sample, at least 50% more reads were classified as XF in each replicate, indicating that the sequencing run could be stopped when the equivalent number of IC reads had accumulated. Based on the sequencing report generated after the demultiplexing step (summary.txt file), we calculated that in this experiment such read number for the IC was obtained after 13 min from run start.

**Figure 3:**
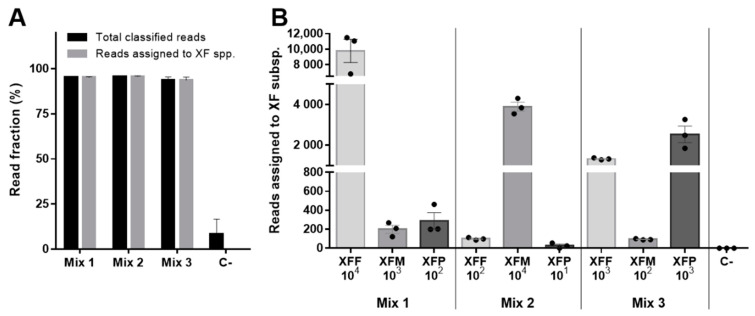
ONT sequencing identifies *X. fastidiosa* (XF) subspecies in complex samples spiked with DNA from all three subspecies. (**A**) For each condition, the bar graph shows the total number of reads with a BLAST hit (total classified reads) and the reads correctly assigned to each XF subspecies. (**B**) For each condition, the chart shows the number of reads assigned to each XF subspecies. Data show median values ± SE for n = 3 technical replicates. XFF = XF subsp. *Fastidiosa*; XFM = XF subsp. *Multiplex*; XFP = XF subsp. *Pauca*; C^−^ = negative control.

To test the ability of the assay to identify multiple XF subspecies in a single complex sample, we applied the same experimental approach used above to detect individual subspecies but mixed the spiked samples so that the three subspecies were present at different concentrations (Table 1). The samples were sequenced in triplicate, generating 131,823 reads in total, among which, 58,312 (44%) could be demultiplexed and 57,698 (44%) were also PASS. We found that the assay correctly identified the presence of XF subspecies at all tested concentrations, demonstrating the ability to discriminate between the three genotypes even in a single sample, which is not possible with standard qPCR-based testing (Figure 3). 

**Figure 4:**
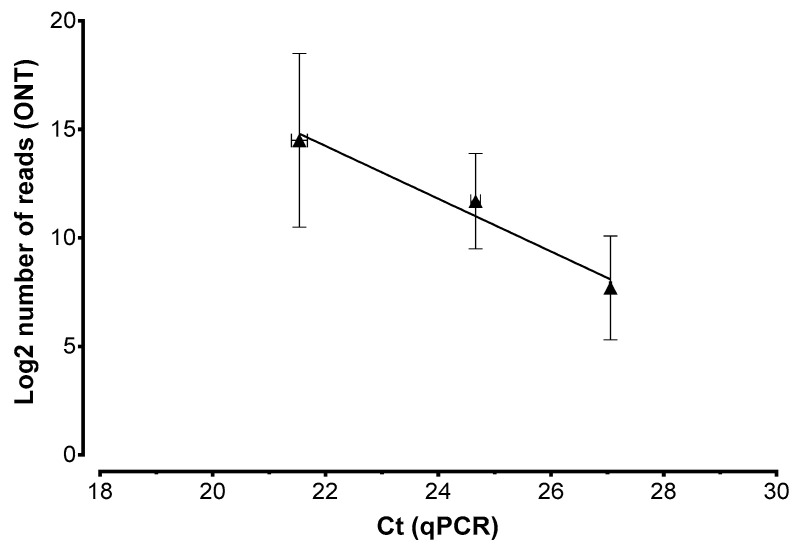
Correlation between ONT and qPCR quantification of *X. fastidiosa* (XF). The number of PASS reads of the three replicates containing *X. fastidiosa* subsp. *fastidiosa* at 10^1^–10^3^ copies/μL (Table 2) correlated with the quantity of XF detected by qPCR (Ct method) in the same samples.

To determine whether the assay produced quantitative data, we prepared samples of oleander DNA spiked with XFF at 10^1^, 10^2^, or 10^3^ genome copies/µL and repeated the analysis as described above. We generated 205,426 total reads, among which, 81,185 (40%) could be demultiplexed and 79,773 (39%) were also PASS. More than 99.9% of the reads were correctly assigned to XFF, with a steady increase in the number of reads from the lowest to the highest concentration we tested (Table 2). Both the total and XFF-assigned reads correlated with the quantification (Ct) obtained by qPCR testing (R^2^ = 0.97), confirming that the assay is indeed quantitative (Figure 4).

## 3. Discussion

The effective management of quarantine pathogens according to international regulations requires fast, sensitive, and cost-effective diagnostic tests that not only detect the pathogen but also distinguish specific genotypes in a quantitative manner [27,37,38]. Current diagnostic testing methods do not address all these needs. We, therefore, developed a new diagnostic assay based on ONT sequencing that achieves the sensitive and quantitative detection of XF in plant samples, including the ability to distinguish between at least three subspecies. 

The new method involves DNA barcoding, which is widely used for species identification and subspecies typing in different fields, including disease monitoring [19]. We amplified a 900-bp portion of the XF genome encompassing part of the HL gene, which is recommended by EPPO international guidelines for the detection of XF and the identification of subspecies [39]. The HL barcode features 30 single-nucleotide variants that allow subspecies identification (Supplementary File S1). Despite the higher error rate of ONT sequencing compared to short-read NGS methods, the application of a robust bioinformatics pipeline resulted in negligible cross-identification even in complex samples containing DNA from XFF, XFM, and XFP, and we recorded no false positives.

The ONT-based assay was also sensitive, resulting in positive results when the bacterial DNA was present at concentrations as low as 10 copies/µL in plant genomic DNA. This is equivalent to the sensitivity of standard qPCR, which has a limit of detection (LOD) of 10 copies/µL [8,36], and to that of the tetraplex qPCR assay, which has a LOD of 4–40 copies/µL. In contrast, the LOD of the end-point PCRs employed in the MLST approach for XF characterization is much higher at 10^2^ copies/µl [35]. Furthermore, although the lowest concentration we tested was 10 copies/µL, we anticipate that the true sensitivity is at least one order of magnitude lower because even the lowest pathogen concentrations generated >10-fold more reads than the negative controls. Most importantly, even when sequencing reads were generated from the negative control samples, they were never assigned to XF, highlighting the discrimination power of NGS-based analysis. In contrast, when using qPCR-based diagnostic assays, the lack of sequence information means that nonspecific amplicons from negative controls are much more difficult to distinguish from samples carrying very low pathogen loads. 

Unlike Illumina and other platforms that use sequencing runs with a predefined loading concentration, time, and output, a unique feature of ONT sequencing is the possibility to stop a run as soon as enough data have accumulated. We showed that an internal control amplicon, present at a low concentration (at or below the assay LOD) and sequenced along with test samples, allowed us to recognize when sufficient data has been generated, avoiding the need for additional sequencing. In addition, since the time required for ONT sequencing can be rather variable depending on flowcell performances, the IC also allows to monitor the production efficiency of each run (or an eventual run failure), thus, guiding the user to extend it until a minimum set of reads has been assigned to the IC. This ensures that sufficient data are produced even from samples with the lowest detectable pathogen loads without the risk of false negative results. The recently introduced “Read Until” feature of ONT sequencing allows the selective sequencing of target DNA molecules as a pool by reversing the voltage across individual nanopores to reject unwanted sequences [40]. This can be exploited to stop the sequencing of samples with a high pathogen load, while favoring those with few or zero pathogen reads “until” a certain amount of data has been produced. The combined use of the internal control and “Read Until” features of ONT diagnostic testing will not only ensure complete and sensitive analysis, but also prevent unnecessary over-sequencing and thus reduce the time and costs associated with pathogen monitoring programs.

The correlation between ONT and qPCR analysis of the same samples indicated that the ONT assay also produces quantitative data. Relative quantitation can, therefore, be achieved by comparing the number of reads assigned to each sample processed in the same sequencing run, whereas absolute quantitation will require the simultaneous sequencing of positive controls with a known concentration to define a standard curve, from which the pathogen copy number can be calculated. Absolute quantification based on nanopore sequencing can also be achieved by integrating unique molecular identifiers (UMIs) in PCR amplicons [41], or by rolling circle amplification (RCA) assays [42]. These enable the grouping of amplification products originating from the same molecule by exploiting the presence a molecule-specific UMI or by producing concatenated amplicons, respectively. However, these protocols are laborious, resource-hungry and require specialized equipment and reagents that are not yet suitable for in-field monitoring [41].

Mixed infections with different XF subspecies are observed in nature [43,44,45] and the same phenomenon is common in other pathogens [46]. The ONT assay easily distinguishes multiple subspecies present in an individual sample, whereas this is much more difficult in MLST assays because mixed infections produce multiple peaks on Sanger chromatograms, which are often mistaken as sequencing errors [45]. While this issue can be bypassed by employing cloning libraries to sequence the different amplicons obtained from samples with mixed infections, the process adds further steps to an already labor-intensive pipeline. The simultaneous identification of XF subspecies can also be achieved by multiplex qPCR with tailored primers and TaqMan probes [35]. However, this only recognizes known genotypes, whereas the ONT approach can also identify novel subspecies. The ability of ONT diagnostic assays to distinguish between subspecies could be further improved by implementing the new Q20+ sequencing chemistry, which is expected to reduce errors to <1%, combined with faster and more accurate base-calling software [47,48]. 

Finally, the ONT-based diagnostic test uses the portable MinION device and can, therefore, be deployed in the field with a minimal package of laboratory equipment. The ease of sample collection and point-of-care testing with the MinION has been demonstrated in multiple field studies [28,29,30,31,32,33,34]. On-site testing will not completely replace the use of central laboratories, but point-of-care analysis can provide rapid preliminary screening results that allow early interventions, such as the quarantine of potentially infectious materials. On-site testing will be enhanced by the latest MinION Mk1C device, which integrates a flow-cell and a GPU node for accelerated base-calling to reduce data processing times. The cost of the full set of equipment required for ONT-based testing is currently ~US$ 7000, which is much lower than the cost of a Sanger sequencer (~US$ 90,000) or a four-color qPCR thermal cycler (~US$ 40,000). Furthermore, although regular flow-cells were used in this study, the sequencing output in each experiment (≤300 Mbp) was far below the full capacity of a standard MinION run (~50 Gbp, as stated by the manufacturer). Therefore, costs can be reduced even further by using Flongles, the disposable and economical ONT flowcells with a reduced capacity of 2.8 Gbp 

In conclusion, we have developed and evaluated a novel ONT-based assay for the detection of a quarantine pathogen in host plant samples. The assay allows simultaneous pathogen detection, quantification, and subspecies identification, with sensitivity and accuracy comparable or superior to standard diagnostic methods. It is also fully compliant with international requirements for the monitoring of quarantine pathogens requiring XF subspecies typing. These features, all included in a one-step assay, together with the portability and low cost of the equipment, and the ability to identify emerging genotypes/variants, pave the way for a new generation of fast, reliable, and robust methods for pathogen detection and surveillance based on ONT sequencing.

## 4. Materials and Methods

### 4.1. Sample Preparation

Test samples were prepared by extracting DNA from asymptomatic *N. oleander* leaves as previously described [11] and mixing 10 ng/µL of this DNA with 10^1^–10^4^ genome copies/µL of XF subspecies (XFF, XFM, and XFP). XFF (DSM 10026) and XFM (DSM 103418) were supplied by the DSMZ (Braunschweig, Germany), whereas XFP (strain ST53) was kindly provided by the Regional Phytosanitary Service of Lombardy. The copy number was calculated based on the genome size of each species and the concentration of DNA extracted from pure cultures. DNA was quantified using a Qubit fluorometer and the Qubit dsDNA BR Assay Kit (Thermo Fisher Scientific, Waltham, MA, USA). The composition of the samples is summarized in Table 1. 

### 4.2. Amplicon Generation

The *Xylella* gene encoding the conserved hypothetical protein HL was amplified using forward primer HL5 (5′-AA GGC AAT AAA CGC GCA CTA-3′), as recommended by the EPPO protocol for XF detection by qPCR combined with a reverse primer designed in this study to anneal ~900 bp downstream, HL-ONT primer (5′- AA GCG CTT TAC CGA CTC AAA-3′). The primers were designed to match conserved regions common to the three XF subspecies, but the amplicon contains 31 single-nucleotide variants that distinguish between them (Supplementary File S1). We amplified 1 ng of DNA in triplicate 50-µL reactions containing 10µM of each primer and LongAmp Taq 2× Master Mix (New England Biolabs, Ipswich, MA, USA) using a MiniOne PCR System (MiniOne Systems, San Diego, CA, USA). The reactions were heated to 95 °C for 2 min followed by 35 cycles of 95 °C for 20 s, 55 °C for 30 s, and 65 °C for 2 min, and a final extension step at 65 °C for 7 min. The products were purified using a 0.9× ratio of AMPureXP beads (Beckman Coulter, Brea, CA, USA) and amplicons were eluted in 15 μL water. The positive control sample was a SARS-CoV-2 cDNA generated using primers 96F (5’-GCCAACAACAACAAGGCCAAAC-3’) and 96R (5’-TAGGCTCTGTTGGTGGGAATGT-3’) from the ARTIC v3 protocol [49]. We amplified 3ng cDNA in triplicate 50 µL reactions containing 10µM of each primer using Q5 High Fidelity DNA Polymerase (New England Biolabs). The reactions were heated to 98 °C for 30 s followed by 30 cycles of 98 °C for 15 s and 65 °C for 5 min. The product was purified using a 0.9× ratio of AMPureXP beads and quantified using a Qubit fluorometer and the Qubit dsDNA BR Assay Kit (Thermo Fisher Scientific).

### 4.3. ONT Sequencing

Sequencing libraries were prepared using the Native Barcoding Amplicons (SQK-LSK109 with barcodes from EXP-NBD104, EXP-NBD114 kits) protocol (ONT, Oxford, UK) and a MiniOne PCR System, with some modifications to conserve reagents. Briefly, 15 μL of each purified amplicon was end-prepped in a 20 μL reaction containing 0.75 μL Ultra II End-prep enzyme mix and 1.75 μL Ultra II End-prep reaction buffer (New England Biolabs). The products were then purified using a 1× ratio of AMPureXP beads and eluted in 10 μL water. Finally, native barcodes were ligated in a 25 μL reaction containing 12.5 μL Blunt/TA ligase Master mix (New England Biolabs) and 2.5 μL barcode mix (ONT). Samples were pooled, purified using a 1× ratio of AMPureXP beads, eluted in 25 μL water, and quantified using Qubit fluorometer and the Qubit dsDNA BR Assay Kit. Only 100–200 fmol of DNA was used for adapter ligation with Quick T4 DNA Ligase (New England Biolabs). The final library was purified using a 0.5× ratio of AMPure beads, quantified using the Qubit dsDNA BR Assay Kit, and ~50 fmol was loaded into a MinION R9.4.1 flowcell for sequencing. We used 3 ng of the internal control (IC) sample and treated it identically to the other samples during library preparation. A total of three flowcells were used in this work, one for each experiment reported respectively in Figure 1, Figure 3, and Table 2. 

### 4.4. Bioinformatic Analysis

Base-calling was applied to raw fast5 files using Guppy v4.2.2 in high-accuracy mode, with parameters “-r -i $FAST5_DIR -s $BASECALLING_DIR --flowcell FLO-MIN106 --kit SQK-LSK109”. Reads were demultiplexed using Guppy v4.2.2 with parameters “-i $BASECALLING_DIR -s $DEMULTIPLEXING_DIR --trim_barcodes --barcode_kits $BARCODE_KITS --require_barcodes_both_ends” and were then filtered by quality (minimum score = 7) using NanoFilt v2.7.1 [50] before conversion to fasta format using seqtk seq (https://github.com/lh3/seqtk accessed on 15 February 2021). Reads from each sample were split into smaller files and processed using Parallel [51], before alignment to the NCBI nt database using nucleotide-nucleotide BLAST 2.9.0+ [52] “-outfmt 6” format. Up to one top hit per read was retained in case the alignment identity and query coverage were higher than 85% and 80%, respectively. Filtered BLAST hits from each file were then merged and a summary file was created containing the number of reads assigned to each taxon, together with the average alignment identity and query coverage. Finally, taxize R package was used to retrieve the full taxonomy for each taxon [53]. The bioinformatic scripts are available at the following URLs: https://github.com/MaestSi/ONT_preprocessing accessed on 15 February 2021 and https://github.com/MaestSi/MetaBlast accessed on 15 February 2021. In Figure 2, the sequencing time points at which the three IC replicates produced on average 50, 100, 150, 200, 250, and 300 reads have been obtained based on the sequencing_summary.txt file. At these time points, the average number of reads and the standard error produced for the three replicates of samples containing 10^1^ XF copies were extracted and plotted using the ggplot2 R package.

### 4.5. Real-Time PCR Assay

The presence of XF in the samples was confirmed using the recommended SYBR green assay [37,54] with primers HL5 and HL6 (5′-GGT TTT GCT GAC TGG CAA CA-3′) to generate a 221 bp amplicon within the HL gene. The reaction mix was prepared as previously described [54] but the total reaction volume was increased to 12 µL. The reaction contained 1× PowerSYBR master mix (Thermo Fisher Scientific), 0.28 µM of each primer, 2 µL of the DNA template, and water up to 12 µL. Triplicate reactions were carried out in a StepOnePlus Real-Time PCR thermocycler (Thermo Fisher Scientific).

## Figures and Tables

**Table 1:** List of samples by name and DNA composition.

Sample	Host DNA		Experimental Spike	
	Host	Concentration	Bacterium	Copies/µL
OXFF_10^1^	NO	10 ng/µL	XFF	10^1^
OXFF_10^2^	NO	10 ng/µL	XFF	10^2^
OXFF_10^3^	NO	10 ng/µL	XFF	10^3^
OXFM_10^1^	NO	10 ng/µL	XFM	10^1^
OXFM_10^2^	NO	10 ng/µL	XFM	10^2^
OXFP_10^1^	NO	10 ng/µL	XFP	10^1^
OXFP_10^2^	NO	10 ng/µL	XFP	10^2^
IC	None	None	SARS-CoV-2, amplicon #96	3 ng
C-	NO	10 ng/µL	None	-
Mix_1	NO	10 ng/µL	XFF, XFM, XFP	10^4^, 10^3^, 10^2^
Mix_2	NO	10 ng/µL	XFF, XFM, XFP	10^2^, 10^4^, 10^1^
Mix_3	NO	10 ng/µL	XFF, XFM, XFP	10^3^, 10^2^, 10^3^

OXFF, OXFM and OXFP: DNA from NO supplmented with XFF, or XFM, XFP respectively. XFF, subsp. fastidiosa; XFM, subsp. multiplex; XFP, subsp. pauca; NO, *Nerium oleander*; IC, internal control; C-, negative control.

**Table 2:** Detection and quantification *X. fastidiosa* (XF) by ONT-based testing. For each condition, the table reports the number of total PASS and demultiplexed reads, reads with a BLAST hit (total classified reads), and reads assigned to *X. fastidiosa* subsp. *fastidiosa* (XFF). Data are means ± SE (n = 3 technical replicates). NO = *N. oleander*; C^−^ = negative control.

Sample DNA	XF Spike Copies/µL	Total PASS & Demultiplexed Reads	Total Classified Reads	Reads Assigned to XFF
NO + XFF	10^3^	23,094 ± 2043	21,752 ± 1859	21,703 ± 1855
NO + XFF	10^2^	3287 ± 157	3092 ± 169	3088 ± 168
NO + XFF	10^1^	205 ± 45	187 ± 45	187 ± 44
NO (C^−^)	0	4 ± 1	0 ± 0	0 ± 0

## Data Availability

Raw genomic sequencing data have been submitted to NCBI GenBank (BioProject no PRJNA792787) under accession number SRR17651910-SRR17651945.

## Supplementary Materials

The following supporting information can be downloaded at: https://www.mdpi.com/article/10.3390/pathogens11020199/s1, Supplementary File S1: HL DNA barcode sequences of XF subspecies.
