# Supplementary material for: Real-Time On-Site Diagnosis of Quarantine Pathogens in Plant Tissues by Nanopore-Based Sequencing
Source: Pathogens. 2022 Feb 2;11(2):199. doi: 10.3390/pathogens11020199 (PMC8876587; doi:10.3390/pathogens11020199)
Supplement: Supplementary file 1 [file pathogens-11-00199-s001.zip › pathogens-1557484-supplementary.pdf]

Supplementary File S1. HL DNA barcode sequences of XF subspecies

```
XFF             TGAGCAAGGCAATAAACGCGCACTATGTCGGACAAATTCTGTGAGGGGGAATCAAATGAC
XFM             ----CAAGGCAATAAACGCGCACTATGTCGGACAAATTCTGTGAGGGGGAATCAAATGAC
XFP             TGAGCAAGGCAATAAACGCGCACTATGTCGGACAAATTCTGTGAGGGGGAATCAAATGAC
                    ********************************************************

XFF             TGCCTTATCTGTGAAACATGCTGCCGATCCAATACGCGGCTTGCAAACACTGACCGTGGA
XFM             TGCCTTATCTGTGAAACATGCTACCGATCCAATACGCGGCTTGCAAACACTGACCGTGGA
XFP             TGCCTTATCTGTGAAATATACTGCCGATCTAATACGCGGCTTGCAAACACTGACCGTGGA
                **************** ** ** ****** ******************************

XFF             AGATATTCGCAGCAATCTAACCAAGGTGTATAGCCGTATCGTTGCTGCCTGCCAAAGTGT
XFM             AGATATTCGCAGCAATCTAACCAAGGTGTATAGCCGTATCATTGCTGCCTGCCAACGTGT
XFP             AGATATTCGCAGCAATCTAACCAAGGTGTATAGCCGTATCATTGCTGCCTGCCAACGTGT
                **************************************** ************** ****

XFF             TGACCGCGATCCCAGCAGCGTGACGCTGTTGCCAGTCAGCAAAACCGCGGATGAAGTGCA
XFM             TAACCGCGATCCCAGCAGCGTGACGCTGTTGCCAGTCAGCAAAACCGTGGATGAAGTGCA
XFP             TGACCGCCATCCCAGCAGCGTGACGCTGTTGCCAGTCAGCAAAACCGTGGATGAAGTGCA
                * ***** *************************************** ************

XFF             TATCCGATTATCCTACGAAGCCGGATGCAGGAAGCTCGGAGAGAACAAAGCTCAGGATGC
XFM             TATCCGATTATCCTACGAAGCCGGATGCAGGAAGCTGGGGGAGAACAAAGCGCAGGATGC
XFP             TATCCGATTATCCTACGAAGCCGGATGCAGGAAGCTGGGGGAGAACAAAGCGCAGGATGC
                ************************************ ** *********** ********

XFF             TTACCACAAGTGGGAAGCGATGAGCGATCTCACTGATCTGTATTGGTCGGTCATTGGGCA
XFM             TTACCACAAGTGGGAAGCGATGAGCGATCTCACTAATCTGCATTGGTCGGTCATTGGACA
XFP             TTACCACAAGTGGGAAGCGATGAGCGATCTCACTGATCTACACTGGTCGGTCATTGGGCA
                ********************************** ****  * ************** **

XFF             CCTGCAAACCAATAAAGCCAAGCTAGTTGCACGGTTTGCCTCGGAATTCCAGGCACTCGA
XFM             CCTGCAAACCAATAAAGCCAAGCTAGTTGCACGGTTTGCCTCGGAATTCCAGGCACTCGA
XFP             CCTGCAAACCAATAAAGCCAAGCTAGTTGCACGGTTTGCCTCGGAATTCCAGGCACTCGA
                ************************************************************

XFF             TAGTTTTCGTGTTGCAGAAGCATTGGAGCGACGTCTTCAGATAGAGGGACGTAGCCTCGA
XFM             TAGTTTGCGTGTTGCAGAAGCATTGGAGCGACGTCTTCAGATAGAGGGACGTAGCCTCGA
XFP             TAGTTTGCGTGTTGCAGAAGCATTGGAACGACGTCTTCAGATAGAGGGACGTAGCCTTGA
                ****** ******************** ***************************** **

XFF             CGTGTTGATTCAAGTCAATACCTCTGGCGAAACAAACAAATACGGATTACCTCCCGAAGA
XFM             CGTGTTGATTCAAGTCAATACCTCCGGTGAAACAAACAAATACGGATTACCTCCCGAAGA
XFP             CGTGTTGATTCAAGTCAATACCTCCGGTGAAACAAACAAATACGGATTACCTCCCGAAGA
                ************************ ** ********************************

XFF             AGTTGTCCGTTTCGTACAGGCACTCTCCGCATATCCAGCGCTTCGGGTACGCGGTTTGAT
XFM             AGTTGTCCGTTTCGTACAGGCACTCTCCGCATATCCAGCGCTTCGGGTACGCGGTTTGAT
XFP             AGTTGTCCGTTTCGTACAGGCACTCTCCGCATATCCAGCGCTTCGGGTACGCGGTTTGAT
                ************************************************************

XFF             GACGTTGGCAATGCTCTCTGGTGACACCTCGCGGGTGCGGCAATGCTTTACACAGCTGCG
XFM             GACGTTGGCGCTGCTCTCTGGTGACACCTCGCGGGTGCGGCAATGCTTTACACAGCTGCG
XFP             GACGTTGGCGCTGCTCTCTGGTGACGCCTCGCGGGTGCGGCAATGCTTTACGCAGCTGCG
                *********  ************** ************************* ********

XFF             CATCCTACGAGATCGCATCCAACAAGCATCACCCCAGGGCCACCACATAACCGAGCTATC
XFM             CATCCTACGAGATCGCATCCAACAAGCATCACCCCAGGGCCACCACATAACCGAGCTATC
XFP             CATCCTACGAGATCGCATCCAACAAGCATCACCCCAGGGCCACCACATAACCGAGCTGTC
                ********************************************************* **

XFF             CATGGGCATGTCAGGAGATTTGGAAATAGCTATAGAAGAAGGGGCGACGGTGGTACGTGT
XFM             CATGGGCATGTCAGGAGATTTGGAAATAGCTATAGAAGAAGGGGCGACGGCGGTACGTGT
XFP             CATGGGCATGTCAGGAGATTTGGAAATAGCTATAGAAGAAGGGGCGACGGTGGTACGTGT
                ************************************************** *********

XFF             AGGACAGGCAATCTACGGTGCACGTGCCCTACCCAACCTCCACTTTCACCATACTGTTTA
XFM             AGGACAGGCAATCTACGGTGCACGTGCCCTACCCAACCTCCACTTGCACCATGCTGTTTA
XFP             AGGACAGGCAATCTACGGTGCACGTGCCCTACCCAACCTCCACTTTCACCATACTGTTTA
                ********************************************* ****** *******

XFF             AGCGCCGCGATTAACTCCTCTCTGAAACTGGTGTTGGTTATAGCAGCGCTTTGAGTCGGT
XFM             AGCGCCGCGATTAACTCCTCTCTGAAACTGGTGTTGGTTATAGCAGCGCTTTGAGTCGGT
XFP             AGCGCCGCGATTAACTCCTCTCTGAAACTGGTGTTGGTTATAGCAGCGCTTTGAGTCGGT
                ************************************************************

XFF             AGAGCG
XFM             AAAGCG
XFP             AAAGCG
                * ****
```
